# Safety and Efficacy of FOLFIRI-3 (Split-Dose Irinotecan) for Unresectable Colorectal Cancer: A Stratified Analysis Based on *UGT1A1* Gene Polymorphisms

**DOI:** 10.3390/cancers18091377

**Published:** 2026-04-26

**Authors:** Gaku Ohira, Hideaki Miyauchi, Toru Tochigi, Tetsuro Maruyama, Koichiro Okada, Atsushi Hirata, Yuko Ikeda, Koichi Hayano, Michihiro Maruyama, Takayuki Ishige, Kazuyuki Matsushita, Kiyohiko Shuto

**Affiliations:** 1 Department of Frontier Surgery, Graduate School of Medicine, Chiba University, 1-8-1 Inohana, Chuo-ku, Chiba 260-8677, Japan; tochigi@chiba-u.jp (T.T.); t.maruyama@chiba-u.jp (T.M.); kookada@chiba-u.jp (K.O.); a.hirata@chiba-u.jp (A.H.); y.ikeda@chiba-u.jp (Y.I.); k-hayano@chiba-u.jp (K.H.); michi-maruyama@chiba-u.jp (M.M.); 2 Department of Surgery, Saisei Hospital, 800-1 Kashiwai, Hanamigawa-ku, Chiba 262-8506, Japan; h.miyauchi@saisei.or.jp; 3 Department of Laboratory Medicine, Chiba University Hospital, 1-8-1 Inohana, Chuo-ku, Chiba 260-8677, Japan; ishige-t@chiba-u.jp (T.I.); kmatsu@faculty.chiba-u.jp (K.M.); 4 Department of Applied Genomics, Kazusa DNA Research Institute, 2-6-7 Kazusa-kamatari, Kisarazu, Chiba 292-0818, Japan; 5 Department of Surgery, Teikyo University Chiba Medical Center, 3426-3 Anesaki, Ichihara, Chiba 299-0111, Japan

**Keywords:** metastatic colorectal cancer, FOLFIRI-3, *UGT1A1* polymorphism, personalized medicine

## Abstract

**Purpose:** *UGT1A1* gene polymorphisms are established risk factors for irinotecan (CPT-11)-induced toxicity. The FOLFIRI-3 regimen, incorporating split-dose CPT-11 administration on Days 1 and 3, was developed to improve safety and efficacy. This study evaluated the clinical outcomes of FOLFIRI-3 stratified by *UGT1A1* genotype. **Methods:** We retrospectively analyzed 147 patients with unresectable metastatic colorectal cancer (mCRC) treated with first-line FOLFIRI-3 between 2005 and 2020 who underwent *UGT1A1* genotyping for *6 and *28 variants. During this period, FOLFIRI-3 was adopted as the standard first-line regimen for all eligible patients at our institution to standardize toxicity management. Patients were classified into three groups: Wild-type (negative for both variants; *n* = 82), Single-heterozygous (SH; *n* = 56), and Homo/Compound heterozygous (HCH; *n* = 8) group. **Results:** The HCH group maintained a relative dose intensity (RDI) of 57.7% with manageable toxicity. While time to treatment discontinuation (TTD) did not differ significantly among groups, overall survival (OS) was significantly longer in the SH group compared to the Wild-type group (Median Survival Time: 30.4 vs. 21.7 months, *p* = 0.0058). **Conclusions:** For high-risk HCH patients, our findings regarding the tolerability of FOLFIRI-3 are strictly preliminary due to the small sample size and require further validation in larger, multi-institutional cohorts. Despite this limitation, this regimen serves as a valuable alternative for high-risk patients and provides superior survival benefits for single heterozygotes, likely due to optimized pharmacokinetics.

## Simple Summary

This retrospective study evaluated the safety and efficacy of the FOLFIRI-3 regimen (split-dose irinotecan) in 147 patients with unresectable metastatic colorectal cancer, stratified by *UGT1A1* polymorphisms. Patients were classified into Wild-type, Single-heterozygous (SH), and Homo/Compound heterozygous (HCH) groups. The HCH group maintained a relative dose intensity of 57.7% with manageable toxicity, supporting the regimen’s safety for high-risk patients. Notably, the SH group demonstrated significantly longer overall survival compared to the Wild-type group (median 30.4 vs. 21.7 months, *p* = 0.0058), likely due to prolonged exposure within the therapeutic window. FOLFIRI-3 serves as a valuable alternative for toxicity management in high-risk patients and offers superior survival benefits for single heterozygotes, highlighting its potential as a tailored strategy in personalized medicine.

## 1. Introduction

Colorectal cancer remains a leading cause of cancer-related mortality worldwide. While the therapeutic landscape is rapidly evolving with the introduction of immunotherapies and targeted agents [1], the combination of fluorouracil (5-FU), leucovorin (LV), and irinotecan (CPT-11)—known as the FOLFIRI regimen—remains a fundamental backbone of first-line chemotherapy for metastatic colorectal cancer (mCRC). Clinical trials, such as those by Douillard et al. [2], the GERCOR study [3], and others [4,5,6,7,8], have demonstrated its survival benefit. However, chemotherapy-induced toxicity poses a persistent challenge in clinical practice [9]. Irinotecan is notably associated with severe, dose-limiting toxicities, particularly neutropenia and delayed diarrhea. The metabolism of irinotecan is heavily dependent on uridine diphosphate glucuronosyltransferase 1A1 (*UGT1A1*). This enzyme glucuronidates SN-38, the active metabolite, into the inactive SN-38 glucuronide (SN-38G). Polymorphisms in the *UGT1A1* gene, specifically *UGT1A1* *28 and *UGT1A1* *6, significantly reduce glucuronidation activity, leading to the accumulation of SN-38 [10,11]. There are substantial ethnic differences in the distribution of these variant alleles [12]. The *28 variant is common in Caucasian populations (30–35%), whereas its frequency is lower in Asians [12]. Conversely, the *6 variant is specific to East Asian populations (15–20%) and is virtually absent in Caucasians [13,14]. In Japanese cancer patients, both *6 and *28 are critical predictors of severe neutropenia [13,15]. Based on these findings and the report by Akiyama et al. [16], it has become common practice in Japan to perform *UGT1A1* polymorphism testing and to consider dose reduction for homozygous or compound heterozygous (HCH) patients, in accordance with the manufacturer’s labeling recommendations [17].

However, dose reduction may compromise antitumor efficacy. To optimize the therapeutic index, researchers have focused on the sequence-dependent synergy between irinotecan and 5-FU. In vitro studies suggested that cytotoxicity is enhanced when irinotecan is administered before 5-FU [18,19,20]. Yet, clinical evaluation in a randomized phase II study revealed that toxicities were exacerbated when 5-FU was followed by irinotecan [21]. These results suggested that while cytotoxicity might be higher, the safety profile is worse with that sequence. Consequently, Mabro et al. initially designed the FOLFIRI-2 regimen, consisting of irinotecan (180 mg/m^2^) administered on Day 3 at the end of the 46-h 5-FU infusion [22]. Although FOLFIRI-2 showed encouraging efficacy in pretreated mCRC, the toxicity profile was unacceptably high, with Grade 3–4 diarrhea in 31%, Grade 3–4 neutropenia in 52%, and febrile neutropenia in 14% of patients. To maintain the therapeutic advantage of administering irinotecan after 5-FU while improving tolerability, the FOLFIRI-3 regimen was developed. FOLFIRI-3 consists of irinotecan (100 mg/m^2^) as a 60-min infusion on Day 1 administered concurrently with leucovorin, followed by a 46-h continuous infusion of 5-FU, and a second infusion of irinotecan (100 mg/m^2^) on Day 3 at the end of the 5-FU infusion [23,24]. This split-dose strategy was adopted to mitigate the severe toxicity observed with the single high-dose administration on Day 3 in the FOLFIRI-2 regimen. We hypothesized that this fractionation strategy, by avoiding a single high-dose administration, may allow for safer administration in high-risk patients with *UGT1A1* polymorphisms and potentially enhance efficacy in intermediate-risk patients. This study analyzes the clinical outcomes of FOLFIRI-3 stratified by *UGT1A1* status.

## 2. Materials and Methods

### 2.1. Study Population (Figure 1)

We retrospectively reviewed patients with unresectable mCRC treated at our institutions between 2005 and 2020. Among 347 patients who underwent systemic chemotherapy during this period, 201 were treated with first-line FOLFIRI-3. During this period, FOLFIRI-3 was our institutional standard to standardize toxicity management. However, alternative regimens (e.g., standard FOLFIRI or FOLFOX) were utilized for patients who declined the split-dose schedule or the logistical requirement of a Day 3 hospital visit, as well as those who found irinotecan-induced alopecia unacceptable or wished to avoid potent cytotoxic agents such as irinotecan or oxaliplatin. Of the 201 patients, 147 who underwent *UGT1A1* genotyping for *6 and *28 were included in this analysis. Since 2010, following the general recommendation for dose reduction in the Japanese drug package insert [17], our institutional policy has been to perform preemptive dose reduction for confirmed HCH patients. In the absence of a standardized national guideline for dose-reduction ratios, our institutional consensus was to initiate irinotecan at a two-level dose reduction (approximately 64% of the standard dose) for HCH patients. The final determination of the regimen and minor adjustments remained at the discretion of the treating physician, who fine-tuned this baseline dose based on the patient’s clinical status (e.g., age, performance status, and prior treatment history). Furthermore, active chemotherapy was generally not recommended for fragile patients with an ECOG Performance Status (PS) of 3. However, for the 7 patients with PS 3 included in this cohort, treatment was initiated only after providing a thorough explanation of the high risk of severe toxicities and obtaining strict informed consent. This decision was ethically justified by the need to respect the strong and explicit requests from the patients and their families to pursue treatment. In all such cases, treatment was carefully initiated at reduced doses with rigorous clinical monitoring to prioritize patient safety.

**Figure 1:**
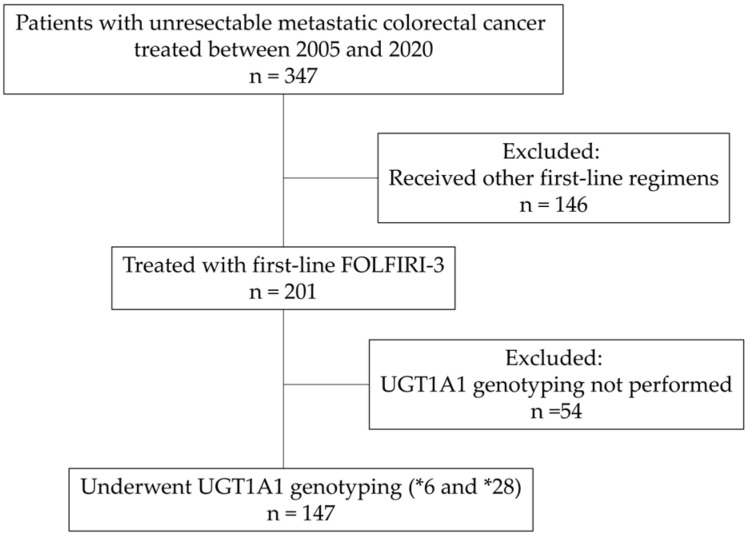
Flow diagram of patient selection.

### 2.2. Treatment Regimen (FOLFIRI-3)

The FOLFIRI-3 regimen followed the protocol described by Mabro et al. [23]:

- Day 1, irinotecan 100 mg/m^2^ as a 1-h infusion, administered concurrently with LV 200 mg/m^2^ as a 2-h infusion, followed by 5FU 2000 mg/m^2^ as a 46-h continuous infusion.
- Day 3, irinotecan 100 mg/m^2^ as a 1-h infusion was repeated, at the end of the 5FU infusion. Cycles were repeated every two weeks, combined with molecular targeted drugs according to standard guidelines [25].

### 2.3. Genotyping and Stratification

The *UGT1A1* genotypes (*6 and *28) were determined by PCR-based melting curve analysis using a previously described method [26]. Stratification was defined as follows: Wild-type (negative for both variant alleles), Single-heterozygous (SH; *1/*6 or *1/*28), and Homo/Compound heterozygous (HCH; *6/*6, *28/*28, or *6/*28). The detailed classification of these genotypes, their zygosity, and assigned metabolic phenotypes are summarized in Table 1.

### 2.4. Statistical Analysis

Overall survival (OS) and time to treatment discontinuation (TTD) were estimated using the Kaplan–Meier method and compared among groups using the log-rank test. To identify independent prognostic factors for OS, variables with a *p*-value < 0.05 in the univariate log-rank test were subsequently included in a multivariate analysis using the Cox proportional hazards model. Hazard ratios (HRs) and 95% confidence intervals (CIs) were calculated. Comparison of the relative dose intensity (RDI) between groups was performed using the Mann–Whitney U test. Adverse events (AEs) were graded according to Common Terminology Criteria for Adverse Events (CTCAE) version 5 [27]. All statistical analyses were performed using JMP Student Edition version 19 (SAS Institute Inc., Cary, NC, USA).

## 3. Results

### 3.1. Patient Characteristics

The genotype distribution was as follows: Wild-type 82 patients (55.8%), SH 56 patients (38.1%), and HCH 8 patients (5.4%). The HCH group included 7 compound heterozygotes (*6/*28) and 1 homozygote (*6/*6). Baseline characteristics showed 15.6% had a Performance Status (PS) ≥ 2 and 23.1% with prior adjuvant chemotherapy. Detailed characteristics are shown in Table 2.

### 3.2. Treatment Efficacy

Regarding the best overall response by Response Evaluation Criteria in Solid Tumor (RECIST) version 1.1 [28], Progressive Disease (PD) was observed in 10 patients (6.8%), Stable Disease (SD) in 45 (30.6%), Partial Response (PR) in 68 (46.3%), and Complete Response (CR) in 7 (4.8%). The objective response rate (ORR) was 51.0%, and the disease control rate (DCR) was 81.6%.

### 3.3. Survival Outcomes

**Figure 2:**
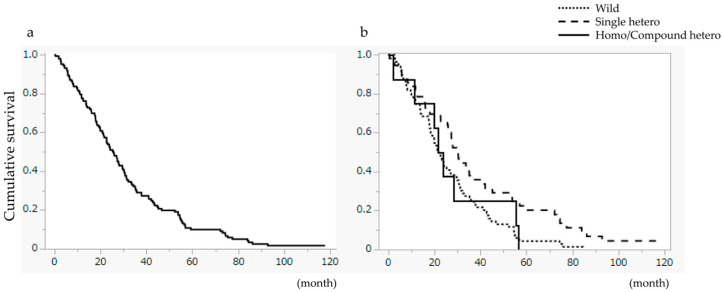
Overall survival (OS). (**a**) Overall survival for all patients. The median survival time (MST) was 25.7 months. (**b**) Comparison of OS based on *UGT1A1* genotype.

**Figure 3:**
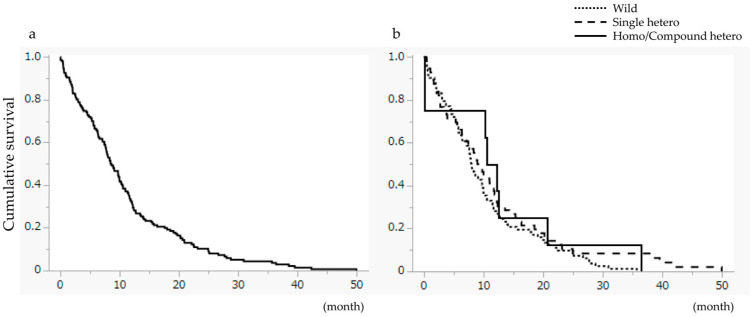
Time to treatment discontinuation (TTD). (**a**) TTD for all patients. The median TTD was 8.6 months. (**b**) Comparison of TTD based on UGT1A1 genotype. No significant difference was observed among the groups.

For the entire cohort, the Median Survival Time (MST) was 25.7 months (Figure 2a). Stratified analysis revealed a significant difference in OS (*p* = 0.0058), with the SH group (MST 30.4 months) performing significantly better than the Wild-type group (MST 21.7 months). The HCH group achieved an MST of 25.7 months (Figure 2b). TTD did not differ significantly among the three groups (Figure 3).

A significant difference was observed among groups (*p* = 0.0171, log-rank test). The MST was 21.7 months in the Wild-type group, 30.4 months in the single heterozygote (SH) group, and 22.8 months in the homo/compound heterozygote (HCH) group. OS was significantly longer in the SH group compared to the Wild-type group (*p* = 0.0058).

Furthermore, univariate and multivariate analyses were performed to identify prognostic factors for OS (Table 3). In the univariate analysis, ECOG PS (0, 1 vs. 2, 3), side of primary tumor (right vs. left), presence of liver metastasis, presence of peritoneal metastasis, *BRAF* status (wild vs. mutant), microsatellite instability (MSS vs. MSI), *UGT1A1* genotype, and main pathology were significantly associated with OS (*p* < 0.05). These significant variables were incorporated into the multivariate Cox proportional hazards model. The multivariate analysis revealed that ECOG PS ≥ 2 (HR 3.66, *p* = 0.0008), presence of liver metastasis (HR 3.16, *p* = 0.0009), mucinous/signet-ring/poorly differentiated histology (HR 2.64, *p* = 0.0264), and *UGT1A1* genotype remained as independent prognostic factors for OS (*p* = 0.0068), confirming the robust survival advantage in the SH group.

### 3.4. Safety and Dose Intensity

Grade 3 or higher AEs occurred in 64 patients (78.1%) in the Wild-type group, 43 patients (78.2%) in the SH group, and 8 patients (100%) in the HCH group. Despite the high incidence in the HCH group, all events were manageable (primarily neutropenia), and no treatment-related deaths occurred (Table 4). The median RDI for irinotecan in the entire cohort was 66.7% (range, 16.6–100%), and an RDI of ≥85% was achieved in 22 cases (15.5%). By group, the median RDI in the Wild-type group was 66.7% (range 16.6–100), in the SH group was 67.3% (range 20.6–95.6), and in the HCH group was 57.7% (range 31.5–94.2). There were no significant differences in RDI among the three groups.

## 4. Discussion

This study re-evaluates the clinical utility of the FOLFIRI-3 regimen in the era of *UGT1A1*-guided personalized medicine. Originally reported by Mabro et al. [23], FOLFIRI-3 enhances safety by splitting the irinotecan dose. While standard FOLFIRI-1 remains the standard practice in Japan due to the logistical burden of Day 3 visits [25], our institutional use of FOLFIRI-3 provided a unique dataset across *UGT1A1* genotypes without selection bias.

Patients with HCH genotypes are at high risk for life-threatening toxicity [13,29,30]. All HCH patients in our study experienced a 100% rate of Grade 3+ AEs, and their median RDI was numerically lower than that of the other groups (57.7%), reflecting the frequent need for dose delays or reductions. However, the absence of treatment-related deaths suggests that the split-dose approach may help avoid fatal outcomes, although severe toxicities remain highly prevalent. This favorable safety profile stands in contrast to the high toxicity previously observed with the single high-dose strategy (FOLFIRI-2) [22], suggesting that fractionating the dose helps mitigate the severity of adverse events. As a real-world retrospective cohort, our analysis included three HCH patients who initially received full doses of irinotecan prior to the availability of genotyping results or established guidelines. While the inclusion of these cases artificially inflated the median RDI of the HCH group (57.7%), the absence of fatal toxicities even in these “worst-case scenarios” underscores a potential safety margin of the split-dose strategy. However, given the 100% incidence of Grade 3 or higher adverse events, FOLFIRI-3 does not eliminate the need for extreme caution, strict monitoring, and appropriate dose reductions in this high-risk population.

While direct comparisons with historical cohorts are limited by differences in patient populations and supportive care over time, the incidences of severe neutropenia (36.1%) and diarrhea (17.0%) in our FOLFIRI-3 cohort did not exceed the rates generally reported in landmark trials of standard FOLFIRI-1 [2,3,4]. However, given the added logistical burden of a Day 3 irinotecan administration, this baseline safety profile alone does not justify the routine adoption of FOLFIRI-3 for all unselected mCRC patients. Instead, we believe the true clinical rationale for the split-dose strategy emerges in the SH subgroup. A novel finding is the superior survival in the SH group (MST 30.4 months) compared to the Wild-type group. Crucially, our multivariate analysis demonstrated that the *UGT1A1* genotype is an independent prognostic factor for OS, even after adjusting for strong clinical variables such as ECOG PS, liver metastasis, and tumor histology. This aligns with clinical reports such as those by Cecchin et al. [31], who observed that *UGT1A1* *28 variant carriers achieved significantly better clinical benefit, response rate, and time to progression (TTP) compared to wild-type patients when treated with irinotecan-based therapy. Cecchin et al. proposed that the mechanism explaining the better outcome of variant carriers could be reduced intratumoral inactivation of SN-38 in variant carriers, as *UGT1A1* is expressed in CRC cells and glucuronidation has been regarded as a mechanism of resistance. Their study ruled out a systemic antitumor effect since *UGT1A1* *28 did not show a strong association with systemic SN-38 AUC. Furthermore, Toffoli et al. [32] and Paoluzzi et al. [33] have shown that *UGT1A1* variants are associated with a higher biliary index (BI) and increased systemic exposure to SN-38. We hypothesize that SH patients might benefit from a “moderate metabolic delay”. In Wild-type patients, rapid glucuronidation may limit tumor exposure to SN-38. In contrast, SH patients likely maintain effective SN-38 levels for a longer duration (increased AUC). This allows them to remain within a “therapeutic window”—maximizing efficacy without exceeding the toxic threshold seen in HCH patients. However, it is important to emphasize that this pharmacokinetic mechanism is highly speculative. Despite this limitation, our clinical survival data suggest that FOLFIRI-3 may be the optimal regimen for SH patients without the need for dose attenuation. Considering the increasing use of intensive triplet regimens (e.g., FOLFOXIRI) and the inherent logistical burden of a Day 3 visit, FOLFIRI-3 is not intended to replace standard FOLFIRI for all unselected patients. Consequently, rather than a universal standard, we propose that FOLFIRI-3 should be positioned as a tailored, highly targeted therapeutic option specifically for SH patients to maximize their survival benefits.

This study has several limitations. First, it is a retrospective analysis from a single center. Second, as a real-world observational study, the cohort inherently includes clinical heterogeneity, such as elderly patients and a small subset with a Performance Status of 3. While this limits the homogeneity of the cohort, our multivariate analysis ensured that these variables were statistically adjusted for when evaluating the primary impact of the *UGT1A1* genotype. Third, the HCH sample size was small (*n* = 8), which severely limits statistical power and the generalizability of conclusions for this high-risk population. Therefore, our findings regarding the safety of FOLFIRI-3 in HCH patients must be considered preliminary and require rigorous validation in larger, multi-institutional cohorts. Fourth, we did not perform a direct internal comparison between FOLFIRI-3 and FOLFIRI-1. Because FOLFIRI-3 was strictly adopted as our institutional standard regimen during the study period, only one patient in our database received standard FOLFIRI-1, precluding any meaningful comparative analysis. Consequently, the true relative advantages of FOLFIRI-3 over FOLFIRI-1 must be evaluated in future prospective randomized studies. Fifth, plasma SN-38 concentrations were not measured directly; our mechanistic explanations are based on the established literature. Therefore, our hypothesis that SH patients benefit from optimal SN-38 exposure remains strictly hypothetical and cannot be definitively confirmed without direct PK measurements. Finally, due to the retrospective design, we could not objectively assess patients’ quality of life (QOL) to fully evaluate the impact of the additional logistical burden imposed by the Day 3 irinotecan administration. However, the acceptable and comparable time to treatment discontinuation (TTD) observed across all genotypes suggests that treatment adherence was reasonably well-maintained in real-world practice.

## 5. Conclusions

Despite the logistical burden, FOLFIRI-3 is a valuable strategy. For HCH patients, while it may help prevent fatal events, the high incidence of severe toxicities and the necessity for dose reductions (as reflected by the lower RDI) indicate that extreme caution and strict monitoring remain essential. Importantly, these findings are preliminary, and the true safety profile and clinical utility for HCH patients must be validated in larger, multi-institutional cohorts. For SH patients, it offers a survival advantage likely due to optimized exposure. We propose FOLFIRI-3 as a tailored option for *UGT1A1* single-heterozygous patients.

## Figures and Tables

**Table 1:** *UGT1A1* genotypes.

Genotype	Zygosity	Enzyme Activity
*1/*1	Wild-type	Normal
*1/*6	Heterozygous	intermediate
*1/*28	Heterozygous	intermediate
*6/*6	Homozygous	Poor
*6/*28	Compound heterozygous	Poor
*28/*28	Homozygous	Poor

**Table 2:** Patient characteristics.

	*n*	%
Age, median (range) (y. o.)	65 (26–84)	
Gender		
Male	79	53.7
Female	68	46.3
ECOG performance status		
0	97	66.0
1	27	18.4
2	16	10.9
3	7	4.8
Side of primary tumor		
Right	49	33.3
Left	98	66.7
Timing of diagnosis		
Primary	100	68.0
Recurrent	47	32.0
Metastatic sites		
Liver	79	53.7
Lung	58	39.4
Peritoneum	42	28.6
Distant lymph node	43	31.3
Primary/Local recurrence	55	37.4
Others	25	17
Prior chemotherapy		
Yes	34	23.1
No	113	76.9
Targeted agents		
Bevacizumab	73	49.7
Cetuximab	12	8.2
Panitumumab	26	17.7
None	36	24.5
Gene status		
*RAS*		
Wild	77	52.4
Mutant	44	30.0
Unknown	26	17.7
*BRAF*		
Wild	88	60.0
Mutant	6	4.1
Unknown	53	36.1
Microsatellite instability		
MSS	61	41.4
MSI-H	2	1.4
Unknown	84	57.1
*UGT1A1*		
Wild (*1/*1)	82	55.8
*6 hetero (*1/*6)	32	21.8
*28 hetero (*1/*28)	24	16.3
*6 homo (*6/*6)	1	0.7
*28 homo (*28/*28)	0	0
Compound hetero (*6/*28)	7	4.8
Main pathology		
Tub/Pap	113	76.9
Muc/Sig/Por	25	17.0
Unknown	9	6.1

Tub, tubular adenocarcinoma; Pap, papillary adenocarcinoma; Muc, mucinous adenocarcinoma; Sig, Signet-ring cell carcinoma; Por, poorly differentiated adenocarcinoma.

**Table 3:** Univariate and multivariate analyses of prognostic factors for overall survival.

	Univariate Analysis		Multivariate Analysis		
	Median Survival Time (Month)	*p* Value	HR	95% CI	*p* Value
Age		0.7035			
<65	27.9				
≥65	22.7				
Gender		0.7706			
Male	27.3				
Female	23.2				
ECOG performance status		<0.0001			0.0008
0, 1	29.6		1.00 (Ref.)		
2, 3	6.6		3.66	1.72–7.80	
Side of primary tumor		0.0110			0.4931
Right	18.7		1.27	0.63–2.53	
Left	28.3		1.00 (Ref.)		
Timing of diagnosis		0.0722			
Primary	21.7				
Recurrent	31.6				
Metastatic sites					
Liver		0.0015			0.0009
Yes	19.8		3.16	1.60–6.25	
No	30.8		1.00 (Ref.)		
Lung		0.3432			
Yes	25.7				
No	26.0				
Peritoneum		0.0072			0.1543
Yes	18.7		1.59	0.84–3.03	
No	28.3		1.00 (Ref.)		
Distant lymph node		0.8711			
Yes	26.0				
No	24.2				
Primary/Local recurrence		0.9177			
Yes	26.0				
No	22.7				
Prior chemotherapy		0.4832			
Yes	27.4				
No	25.4				
Targeted agents		0.9902			
Yes	23.3				
No	29.6				
Gene status					
*RAS*		0.6152			
Wild	26.0				
Mutant	22.7				
*BRAF*		0.0229			0.0954
Wild	22.7		1.00 (Ref.)		
Mutant	10.0		3.10	0.82–11.7	
Microsatellite instability		0.0043			0.1838
MSS	21.7		1.00 (Ref.)		
MSI	6.13		3.83	0.53–27.9	
*UGT1A1*		0.0272			0.0068
Wild	22.7		1.00 (Ref.)		
SH	29.7		0.41	0.21–0.78	
HCH	22.8		1.58	0.30–8.24	
Main pathology		0.0206			0.0264
Tub/Pap	26.0		1.00 (Ref.)		
Muc/Sig/Por	17.5		2.64	1.12–6.21	

**Table 4:** Adverse Events (CTCAE Grade ≥ 3).

	*n*	%
Non-hematological		
Nausea	9	6.1
Vomiting	4	2.7
Diarrhea	25	17.0
Stomatitis	1	0.7
Anorexia	33	22.4
Infusion reaction	1	0.7
Dry skin	3	2.0
Paronychia	2	1.4
Hematological		
Leukopenia	22	15
Neutropenia	53	36.1
Anemia	6	4.1
Febrile neutropenia	4	2.7

## Data Availability

The data are not publicly available due to ethical restrictions.

## Abbreviations

The following abbreviations are used in this manuscript:

5-FU	Fluorouracil
AE	Adverse event
BI	Biliary index
CPT-11	Irinotecan
CR	Complete response
CTCAE	Common Terminology Criteria for Adverse Events
DCR	Disease control rate
ECOG	Eastern Cooperative Oncology Group
HCH	Homo/compound heterozygote
LV	Leucovorin
mCRC	Metastatic colorectal cancer
MSI	Microsatellite instability
MSS	Microsatellite stable
MST	Median survival time
ORR	Objective response rate
OS	Overall survival
PD	Progressive disease
PR	Partial response
PS	Performance status
RDI	Relative dose intensity
RECIST	Response Evaluation Criteria in Solid Tumors
SD	Stable disease
SH	Single heterozygote
SN-38G	SN-38 glucuronide
TTD	Time to treatment discontinuation
TTP	Time to progression
UGT1A1	Uridine diphosphate glucuronosyltransferase 1A1
